# Presence of *Porphyromonas gingivalis* in esophagus and its association with the clinicopathological characteristics and survival in patients with esophageal cancer

**DOI:** 10.1186/s13027-016-0049-x

**Published:** 2016-01-19

**Authors:** Shegan Gao, Shuoguo Li, Zhikun Ma, Shuo Liang, Tanyou Shan, Mengxi Zhang, Xiaojuan Zhu, Pengfei Zhang, Gang Liu, Fuyou Zhou, Xiang Yuan, Ruinuo Jia, Jan Potempa, David A. Scott, Richard J. Lamont, Huizhi Wang, Xiaoshan Feng

**Affiliations:** Henan Key Laboratory of Cancer Epigenetics, Cancer Institute, The First Affiliated Hospital, and College of Clinical Medicine of Henan University of Science and Technology, Luoyang, 471003 China; Department of Oncology, Anyang People’s Hospital, Anyang, 471500 China; Department of Microbiology, Faculty of Biochemistry, Biophysics, and Biotechnology, Jagiellonian University, Krakow, Poland; Department of Oral Immunology and Infectious Diseases, University of Louisville School of Dentistry, Room 263D, 501 South Preston Street, Louisville, KY 40202 USA; Department of Oncology, Henan University of Science and Technology, 24 Jinghua Road, Jianxi Qu, Luoyang, 471500 Henan China

**Keywords:** *Porphyromonas gingivalis*, ESCC, Lys-gingipain, 16S rDNA, Oral pathogen, Differentiation, Metastasis, Overall survival rate, Prognoses

## Abstract

**Background:**

Mounting evidence suggests a causal relationship between specific bacterial infections and the development of certain malignancies. However, the possible role of the keystone periodontal pathogen, *Porphyromonas gingivalis*, in esophageal squamous cell carcinoma (ESCC) remains unknown. Therefore, we examined the presence of *P. gingivalis* in esophageal mucosa, and the relationship between *P. gingivalis* infection and the diagnosis and prognosis of ESCC.

**Methods:**

The presence of *P. gingivalis* in the esophageal tissues from ESCC patients and normal controls was examined by immunohistochemistry using antibodies targeting whole bacteria and its unique secreted protease, the gingipain Kgp. qRT-PCR was used as a confirmatory approach to detect *P. gingivalis* 16S rDNA. Clinicopathologic characteristics were collected to analyze the relationship between *P. gingivalis* infection and development of ESCC.

**Results:**

*P. gingivalis* was detected immunohistochemically in 61 % of cancerous tissues, 12 % of adjacent tissues and was undetected in normal esophageal mucosa. A similar distribution of lysine-specific gingipain, a catalytic endoprotease uniquely secreted by *P. gingivalis*, and *P. gingivalis* 16S rDNA was also observed. Moreover, statistic correlations showed *P. gingivalis* infection was positively associated with multiple clinicopathologic characteristics, including differentiation status, metastasis, and overall survival rate.

**Conclusion:**

These findings demonstrate for the first time that *P. gingivalis* infects the epithelium of the esophagus of ESCC patients, establish an association between infection with *P. gingivalis* and the progression of ESCC, and suggest *P. gingivalis* infection could be a biomarker for this disease. More importantly, these data, if confirmed, indicate that eradication of a common oral pathogen could potentially contribute to a reduction in the overall ESCC burden.

**Electronic supplementary material:**

The online version of this article (doi:10.1186/s13027-016-0049-x) contains supplementary material, which is available to authorized users.

## Background

Since the discovery that *Helicobacter pylori* plays a causative role in gastric adenocarcinoma, multiple other associations between specific bacteria and cancer have been reported [1, 2], including *Salmonella typhi* with gall bladder cancer [3], *Streptococcus bovis* with colon cancer [4], *Chlamydophila penumoniae* with lung cancer [5], *Bartonella species* with vascular tumor formation [6], *Propionibacterium acnes* with prostate cancer [7], and *Escherichia coli* with colon cancer [8]. Esophageal cancer is the eighth most frequent tumor and sixth leading cause of cancer death worldwide. Whereas the majority of cases occur in Asia, particularly in central China, recent data suggest that the frequency of new cases is rising in Western Europe and the USA [9, 10]. Two major histological subtypes of esophageal cancer have been identified including squamous cell carcinoma (ESCC), which is more common in developing countries, and adenocarcinoma, which is more common in developed nations [11]. Esophageal cancer is characterized by difficulty of early diagnosis, rapid development and high mortality. Therefore, there is a considerable need to better understand causative agents in order to reduce the incidence and mortality of this disease. Like most cancers, a plethora of risk factors including age, gender, heredity, gene mutation, chemical exposure, and diet have been reported for esophageal cancer [12, 13].

A potential contribution of microbes to the development of esophageal cancer is beginning to emerge. Pei et al. reported that *Streptococcus, Prevotella* and *Veillonella* are the most prevalent genera detected in esophageal biopsies [14, 15]. Yang et al. have classified the esophageal microbiota into two subtypes: the *Streptococcus*-dominated type I microbiome, which is mainly associated with a normal esophagus, and the type II microbiome, in which Gram-negative anaerobes predominate, which is associated with Barrett’s esophagus (BE) and esophagitis [16]. A significant association between the inhabitants of the upper digestive tract microbiota and esophageal squamous dysplasia, a precursor lesion of esophageal squamous cell carcinoma, has also been reported [17]. While there are several phylum-wide studies on the esophageal microbiota and the possible associations with reflux esophagitis, Barrett’s esophagus, and esophageal squamous dysplasia, there has been no research on the esophageal microbiota in patients suffering from ESCC, especially at species level, let alone the possible association of these bacteria with the development of ESCC.

The microbiome in chronic and severe manifestations of periodontal disease is enriched for Gram-negative anaerobic bacteria. Among these, *Porphyromonas gingivalis* is a keystone oral pathogen which can invade epithelial cells, and interfere with host immune responses and the cell cycle machinery [18–20]. Epidemiological studies have demonstrated that periodontal diseases and tooth loss are significantly associated several cancers such as oral cancer, gastric cancer, and pancreatic cancer and may even relate to survival [20–24]. *P. gingivalis*-mediated immune evasion, apoptosis inhibition, carcinogen conversion, induction of MMP-9 and dysbiosis of the oral microbiota have all been posited as pro-tumorigenic mechanisms in the context of oral squamous cell carcinoma [20, 25]. Since esophageal squamous cells are histologically similar to oral squamous cells and esophageal infection arising from the oral niche is highly plausible, we hypothesized that *P. gingivalis* may be associated with ESCC. We set out to test this hypothesis using 100 ESCC subjects and 30 normal matched controls.

## Results

### Immunohistochemical detection of *P. gingivalis* presence is more common in ESCC

**Fig. 1:**
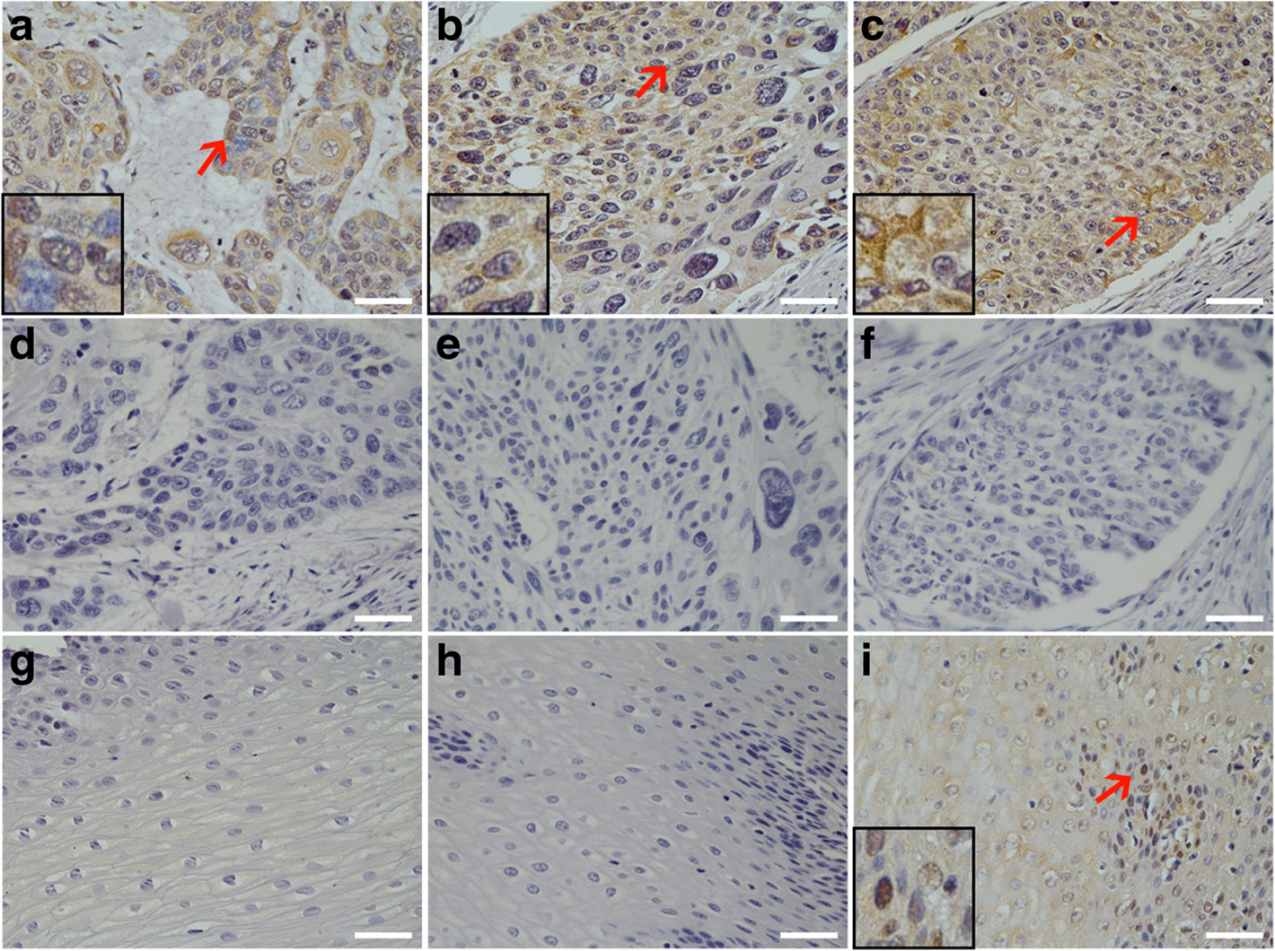
Immunohistochemical detection of *P. gingivalis* in normal esophageal mucosa, and cancerous and adjacent tissues of ESCC. **a**, **b**, and **c** are representative images of *P. gingivalis* in well differentiated (**a**), moderately differentiated (**b**), and poorly differentiated (**c**) ESCC tissues. Pre-immune rabbit IgG was used as a control to detect the serial tissue sections from the same paraffin-embedded tissue block of: (**d**) well differentiated-; (**e**) moderately differentiated-; and (**f**) poor differentiated ESCC. **g** Normal esophageal mucosa stained with anti-*P. gingivalis* anti-serum; (**h**) and (**i**) are the representative negative/positive images of *P. gingivalis* immunostaining in the adjacent cancerous tissues. 20× magnification; scale bar = 50 μm; miniatures in the left corners amplify the area with *red arrows*

As shown in Fig. 1, *P. gingivalis* was detected in cancerous and adjacent esophageal mucosa, but not healthy mucosa. Furthermore, *P. gingivalis* infection was more common in cancerous tissue (61 %) than adjacent tissue (12 %) or normal control tissue (0 %) (both *p* < 0.01, Table 1). *P. gingivalis* was primarily immunolocalized to the epithelial cell cytoplasm but bacterial antigens were occasionally present in nuclei.

**Table 1:** Presence of *P. gingivalis* and Lys-gingipain (Kgp) detected by specific antibodies in normal esophagus mucosa, cancerous and adjacent tissues of ESCC

Factors	Pg positive cases (%)	KGP positive cases (%)
ESCC samples (*n* = 100)	61 (61)*	66 (66)*
Adjacent normal tissues (*n* = 100)	12 (12)*	17 (17)*
Normal esophageal mucosa (*n* = 30)	0 (0)*	0 (0)*

*p < 0.01

### Expression of *P. gingivalis* lysine-gingipain (Kgp) is more common in ESCC

**Fig. 2:**
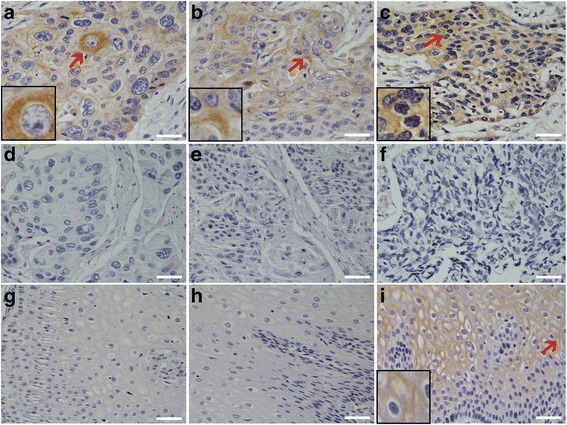
Immunohistochemical detection of Lys-gingipain (Kgp) in normal esophagus mucosa, cancerous and adjacent tissues of ESCC. **a**, **b**, and **c** are representative images of Lys-gingipain in well differentiated (**a**), moderately differentiated (**b**), and poorly differentiated (**c**) ESCC tissues. Normal mouse IgG was used as a control to detect the serial tissue sections from the same paraffin-embedded tissue block of (**d**) well differentiated-; (**e**) moderately differentiated-; and (**f**) poorly differentiated ESCC. **g** Normal esophageal mucosa stained with anti-Lys-gingipain antibody; (**h**) and (**i**) are the representative negative/positive images of Lys-gingipain immunostaining in the adjacent cancerous tissues. 20× magnification; scale bar = 50 μm; miniatures in the left corners amplify the area with *red arrows*

To corroborate the presence of *P. gingivalis* antigens in esophageal epithelium, we next used a Kgp-specific antibody. As shown in Fig. 2, the expression pattern of Kgp reflected that of the whole cell antigens as above, being primarily expressed in the epithelial cytoplasm but occasionally in nuclei, and expressed at significantly high levels in cancerous tissue (66 %), as compared with adjacent (17 %) or healthy tissues (0 %) (both *p* < 0.01, Table 1).

### *P. gingivalis* 16S rDNA is more frequent in ESCC

To control for false positives due to possible cross-reaction of antibodies, we next employed qRT-PCR to examine the presence of 16S rDNA in fresh esophageal tissue specimens. All esophageal samples were positive for the presence of bacteria, as determined using universal 16S rDNA primers (Additional file 1: Figure S1). Similar to the staining pattern with antibodies to the whole cells and to Kgp, *P. gingivalis* 16S rDNA was present in significantly more frequently in the cancerous tissues (71 %) than adjacent (12 %) or normal esophagus mucosa (3.3 %; both *p* < 0.001), as presented in Table 2.

**Table 2:** PCR-detected expression of *P. gingivalis* in normal esophagus mucosa, cancerous and adjacent tissues of ESCC

Factors	Positive (%)	Negative (%)	*p* value
ESCC samples (*n* = 100)	71 (71)	29 (29)	<0.0001
Adjacent normal tissue (*n* = 100)	12 (12)	88 (88)	
Normal esophageal mucosa (*n* = 30)	1 (3.3)	29 (96.7)	

### Comparison of different methods for the detection of *P. gingivalis* presence

We next analyzed the correlation between the expression of *P. gingivalis* whole antigens and Kgp, and the concordance between the results of immunohistochemistry (IHC) and real time qPCR. Of the 100 esophageal cancerous tissue specimens that were analyzed in this study, 59 % of samples were positive for immunohistochemical staining for both *P. gingivalis* cells and Kgp enzyme (Table 3). The level of *P. gingivalis* whole cell staining was found to significantly correlate with the level of the Kgp staining in cancerous tissue of ESCC patients (*p* <0.0001; Pearson’s contingency coefficient = 0.630). Moreover, we compared the results of IHC and qRT-PCR for the presence of *P. gingivalis* to determine the agreement between these two different methods. The data showed that the average percentage of cancerous tissues positively stained with anti-*P. gingivalis* antibody in the qRT-PCR positive tumors was significantly higher than in the qRT-PCR negative tumors (84.5 % versus 3.4 %; *p* <0.0001). There was only one case with IHC scores of 2 that was qRT-PCR negative. However, we found eleven cases with IHC scores of lower than 2 that were qRT-PCR positive (Table 4), suggesting our IHC standard is higher and qRT-PCR is more sensitive for the detection of *P. gingivalis* in cancerous tissue. In current study, IHC scores with whole *P. gingivalis* antigen equal or more than 2 were *P. gingivalis*-positive. The sensitivity and specificity of IHC were 84.5 and 96.6 %, respectively. The concordance rate was 88 % (kappa = 0.736; *p* < 0.001) between IHC and qRT-PCR (Table 4). When we re-examined the eleven cases which were IHC negative and qRT-PCR positive, we found 7 of them staining with an IHC score close to 2, suggesting the positive criteria of IHC is critical for the agreement of these two methods. If we set the samples with IHC scores more than 1 as *P. gingivalis* positive, the sensitivity and specificity of IHC would be 94.3 and 96.6 %, respectively (Kappa = 0.882; *p* < 0.001, Additional file 2: Table S1), showing excellence concordance between IHC and qRT-PCR for the detection of *P. gingivalis* infection in the cancerous tissues of ESCC patients.

**Table 3:** Pearson’s contingency coefficient analysis of the correlation between the levels of immunohistochemical staining of *P. gingivalis* and KGP in cancerous tissue of ESCC patients

Antigens	KGP expression		R value	*p* value
	+	-		
Pg (+)	59	2	0.630	<0.0001
Pg (−)	7	32		
Total	66	34		

*Data presented as the number of patients. *P* < 0.0001; Pearson’s contingency coefficient =0.630

**Table 4:** Concordance between the immunohistochemistry with *P. gingivalis* whole cell antibodies and qPCR of *P. gingivalis* 16S rDNA in the cancerous tissue from patients with ESCC

Approaches	PCR expression		Kappa value	*p* value
	+	-		
IHC (+)	60	1	0.736	<0.0001
IHC (−)	11	28		
Total	71	29		

*Kappa value >0.7 excellent; 0.4–0.7, good; <0.4, poor agreement

### *P. gingivalis* infection is positively correlated to clinicopathologic characteristics of ESCC

Since an association between *P. gingivalis* infection and ESCC had been demonstrated, we next sought to determine if the presence of *P. gingivalis* antigens is associated with the progression of esophageal cancer. Pathological information of the ESCC patients is presented in Table 5. While the presence of *P. gingivalis* was not significantly associated with age, gender, or smoking history of the patients, the presence of *P. gingivalis* was positively related to differentiation, lymph node metastasis and the TNM stage of ESCC (*p* < 0.05). A positive immunohistochemical signal for *P. gingivalis* was 90 % in the poorly differentiated tissues, which was significantly higher than that of well or moderately differentiated samples (*p* < 0.05) (Table 5). Moreover, the percentage of *P. gingivalis* infected lymph node metastasis samples was 84.2 %, statistically higher than that of non-metastatic group (46.8 %; *p* < 0.05) (Table 5). Similar relationships between *P. gingivalis*-derived Kgp expression, cancer cell differentiation and metastasis were observed (Table 5). Additionally, the presence of *P. gingivalis* was closely related to the TNM stage of ESCC. Late stage ESCC tissues were more likely to be positive for whole *P. gingivalis*-derived antigens (87.5 %) or Kgp (84.4 %) than early stage ESCC (48.5 and 57.4 %, respectively; *p* < 0.05). Taken together, these results reveal that *P. gingivalis* infection is positively correlated with poor differentiation, severe lymph node metastasis and stage of ESCC, suggesting that *P. gingivalis* could be a novel etiologic agent and potential prognostic indicator of this important malignant disease.

**Table 5:** Association between the presence of *P. gingivalis* or Lys-gingipain and the clinicopathologic features of ESCC patients

Factors	Pg positive cases (%)	KGP positive cases (%)
ESCC samples (*n* = 100)	61(61)**	66(66)**
Adjacent normal tissues (*n* = 100)	12(12)**	17(17)**
Normal esophageal mucosa (*n* = 30)	0(0)**	0(0)**
Gender		
Male (*n* = 70)	45(64.3)	48(68.6)
Female (*n* = 30)	16(53.3)	18(60.0)
Age (years)		
≤60 (*n* = 39)	24(61.5)	23(59.0)
>60 (*n* = 61)	37(60.7)	43(70.5)
Smoking history		
Smoking (*n* = 45)	31(68.9)	32(71.1)
Non-smoking (*n* = 55)	30(54.5)	34(61.8)
Differentiation		
Well (*n* = 22)	8(36.4)*	10(45.5)*
Moderate (*n* = 58)	35(60.3)*	39(67.2)*
Poor (*n* = 20)	18(90.0)*	17(85.0)*
Lymph node metastasis		
Positive (*n* = 38)	32(84.2)*	31(81.6)*
Negative (*n* = 62)	29(46.8)*	35(56.5)*
TNM stage		
I+ II (*n* = 68)	33(48.5)*	39(57.4)*
III (*n* = 32)	28(87.5)*	27 (84.4)*

“**”and “*”indicate *p* < 0.01 and *p* <0.05, respectively

### *P. gingivalis* infection is negatively correlated with overall ESCC survive rate

**Fig. 3:**
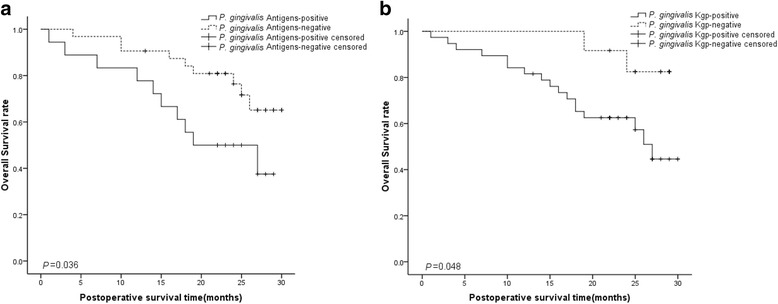
Kaplan-Meier Survival Analysis of the relationship between the presence of *P. gingivalis* and the expression of Lys-gingipain and the overall survival rate of ESCC patients. Higher expression of the *P. gingivalis* whole cell antigens (**a**) or Lys-gingipain (**b**) were both positively related with poorer overall survival rate of ESCC patients. The *p* value is 0.036 (**a**) and 0.048 (**b**) respectively

In order to assess the potential consequences of *P. gingivalis* infection on ESCC patients, we next compared the overall cumulative survival rate in ESCC patients with and without *P. gingivalis* infection. A total of 100 patients were followed up for survival analysis over a period of 30 months (Table 6). Because of the limited follow-up time, the survival rate of both groups was greater than 40 % and the median survival time could not be calculated. However, the mean survival time for patients with positive *P. gingivalis* antigen expression was 20.139 months, significantly lower than that of *P. gingivalis* negative group (25.971 months) or all patients (23.981 months) (both *p* < 0.05) (Table 6). Similar results were found when Lys- gingipain expression were examined, with mean survival times of 22.475, 27.078, and 23.981 months for positive, negative and all patients group, respectively (6). Furthermore, Kaplan-Meier analysis showed that the difference between negative and positive *P. gingivalis* presence was significant for overall survival both for *P. gingivalis* positive staining (*n* = 100, *p* = 0.036) (Fig. 3a) and Kgp positive expression (*n* = 100, *p* = 0.048) (Fig. 3b).

**Table 6:** Means and medians for the survival time (months) of ESCC patients with positive or negative expression of *P. gingivalis* and Lys-gingipain

Factors	Group	Mean^a^				Median^a^				*p* value
		95 % Confidence interval								
		Est.	Std. error	Lower bound	Upper bound	Est.	Std. error	Lower bound	Upper bound	
Pg	Positive	20.139	2.238	15.752	24.526	19.000	5.874			
	Negative	25.971	1.264	23.493	28.449					
	Overall	23.981	1.225	21.580	26.382					0.036
KGP	Positive	22.475	1.507	19.521	25.430	27.000	1.648	23.770	30.230	
	Negative	27.708	0.874	25.996	29.421					
	Overall	23.981	1.225	21.580	26.382					0.048

^a^Estimation is limited to the largest survival time; “Est.” and “Std.” are the abbreviations of “estimated” and “standard” respectively

## Discussion

To the best of our knowledge, the composition and potential role of the esophageal microbiota in the patients suffering from ESCC have not been investigated. Using three complementary approaches, we have established that antigens and DNA from *P. gingivalis,* a periodontal pathogen*,* can be detected in the epithelium of the esophagus of ESCC patients. The intensity of expression of whole *P. gingivalis* antigen, its unique protease Lys-gingipain, and detection of *P. gingivalis*-specific16S rDNA were all significantly higher in the cancerous tissue of ESCC patients than in the surrounding tissue or normal control sites. Moreover, our analysis indicates that the presence of *P. gingivalis* correlates with multiple clinicopathologic factors, including cancer cell differentiation, metastasis, and overall survival ESCC rate. These findings provide the first direct evidence that *P. gingivalis* infection could be a novel risk factor for ESCC, and may also serve as a prognostic biomarker for this prevalent cancer.

A number of aspects of the interaction of *P. gingivalis* with host epithelial cells provide a plausible molecular basis for potential *P. gingivalis*-mediated carcinogenesis [20, 25, 26]. First, chronic inflammation *per se* is associated with the development of cancer [27], and, for example, prolonged IL-6 signaling and STAT3 activity is known to be pro-tumorogenic [28, 29]. In this regard, both our group and others have demonstrated that *P. gingivalis* activates JAK2 and GSK3β pathways, thus increasing the production of IL-6 in epithelial cells [28, 30]. Secondly, *P. gingivalis* can promote tumorigenesis by secreting a nucleoside diphosphate kinase (NDK). NDK from *P. gingivalis* antagonizes ATP activation of P2X_7_ receptors, and thus reduces IL-1β production from epithelial cells [31]. Since IL-1β is critical for priming IFNγ-producing, tumor–antigen-specific CD8^+^ T cells, NDK from *P. gingivalis* could promote the immune evasion of tumor cells [20]. Moreover, NDK-mediated degradation of ATP also suppresses apoptosis dependent on ATP activation of P_2_X7 receptors [32]. Thirdly, *P. gingivalis* inhibits epithelial cell apoptosis by a number of mechanisms, including activation of Jak1/Akt/Stat3 [33, 34], enhancing the Bcl2 (antiapoptotic): Bax (proapoptotic) ratio, blocking the release of the apoptosis effector cytochrome c, and the activation of downstream caspases [35]. Moreover, *P. gingivalis* can upregulate microRNAs, such as miR-203, which suppress apoptosis in primary gingival epithelial cells [36]. In concert with suppression of apoptosis, *P. gingivalis* can accelerate progression through the cell cycle by manipulation of cyclin/CDK (cyclin-dependent kinase) activity and reducing the level of the p53 tumor suppressor [37]. Lastly, in oral squamous cell carcinoma (OSCC) cells, *P. gingivalis* promotes cellular migration through activation of the ERK1/2-Ets1, p38/HSP27, and PAR2/NF-κB pathways to induce pro-matrix metalloproteinase (MMP)-9 expression [25]. Apart from all the above, another possible mechanism for *P. gingivalis* induced carcinogenesis is the metabolism of potentially carcinogenic substances. For example, *P. gingivalis* converts ethanol into its carcinogenic derivative, acetaldehyde, to levels capable of inducing DNA damage, mutagenesis and hyperproliferation of the epithelium [38, 39], which could help explain the epidemiological evidence associating heavy drinking and development of some cancers [20].

While it is possible that *P. gingivalis* infection initiates or is a co-factor in the transformation of esophageal epithelial cells, the possibility that cancer tissues represent a preferred microenvironment for *P. gingivalis* cannot be excluded. Thus, while our results reveal a positive association between infection with *P. gingivalis* and the progression of ESCC, *P. gingivalis* is not yet established as a novel etiological agent or co-factor of ESCC. Should *P. gingivalis* prove to cause ESCC, the implications are enormous. It would suggest (i) that improved oral hygiene might reduce ESCC risk, (ii) that screening for *P. gingivalis* in dental plaque may identify susceptible subjects, and that (iii) antibiotic use, or other anti-bacterial strategies, may prevent ESCC progression. Should the clear association between *P. gingivalis* infection and ESCC turn out to be better explained by physiological conditions inside ESCC cells being more amenable to *P. gingivalis* survival and growth, this would imply that attenuated *P. gingivalis* or non-pathogenic bacteroidetes strains that contain eukaryotic lysins may represent a novel and effective therapeutic approach for ESCC. In this regard, several studies have attempted to take advantage of the oxygen-limited conditions present in malignant cells to develop anaerobic, non-pathogenic bacteria for the delivery of cancer cell cytolysins [40]. These include *Clostridium novyi* for the treatment of melanoma and modified *Bifidobacterium longum* carrying 5-fluorocytosine for the treatment of breast cancer [41–43]. Hence, further studies to determine if *P. gingivalis* infection promotes the initiation and progression of ESCC are required.

Finally*,* colonization by *P. gingivalis* promotes the conversion of a symbiotic to a dysbiotic of oral microbiota, a process considered critical for the progression of periodontal disease [44]. Dysbiosis of the microbiota in the esophagus could potentially cause or exacerbate the severity of esophageal disorders [18]. Thus, a further possibility to be tested is that esophageal infection with *P. gingivalis* leads to shift in the microbiome involved in the development of esophageal cancer.

## Conclusion

In summary, we have established that *P. gingivalis* molecules are present in the epithelium of the esophagus of ESCC patients and provide the first direct evidence of a positive correlation between *P. gingivalis* infection, ESCC severity and poor prognosis. These findings demonstrate for the first time that *P. gingivalis* infects the epithelium of the esophagus of ESCC patients, establish the association between the infection of *P. gingivalis* and the progression of ESCC, and suggest *P. gingivalis* infection could be a biomarker for this disease. More importantly, these data, if confirmed, indicate that eradication of an oral pathogen could potentially contribute to a reduction in the overall ESCC burden.

## Methods

The study was approved by the Institutional Review Board of the University of Henan University of Science and Technology (HUST).

### Patients and human tissue

One hundred patients with ESCC who underwent esophagectomy surgery from 2010 to 2014 at the First Affiliated Hospital of Henan University of Science and Technology and Anyang people’s hospital were investigated in this study. Adjacent tissue samples were obtained 3 cm distant to cancerous tissue. Thirty additional specimens were randomly selected during endoscopic examination from biopsy, and confirmed histologically as normal esophagus mucosa. Demographics (sex and age) and clinicopathologic features (differentiation status, lymphatic invasion, lymph node metastasis, TNM stage) were obtained from medical records. A smoker was defined as someone who had smoked one cigarette or more per day for at least 1 year. Overall survival rates were determined over 30 months.

### Immunohistochemistry

Tissues were fixed in formalin and then embedded in paraffin. Serial sections of 4 mm thickness were prepared and deparaffinized by submersion in three separate concentrations of ethanol (100, 95, and 70 %), and rinsing continuously in distilled water for 5 min. Antigen retrieval was performed by incubating slides in antigen retrieval Citra plus solution (BioGenex, San Ramon, USA), according to the manufacturer’s instructions. Slides were blocked 1.5 % normal goat serum (Vector Laboratories, Burlingame, USA) for 30 min. Polyclonal rabbit anti-*P. gingivalis* 33277 [45] and monoclonal mice anti-Lys-gingipain (Kgp) (15C8G5E6C2) antibodies [46] were utilized for the detection of *P. gingivalis*. Pre-immune rabbit IgG and normal mouse IgG was used as a negative control. Primary antibodies were incubated with tissue sections (anti-whole cells 1:1000 dilution; anti-Lys-gingipain 1:500 dilution) for 12 h, 4 °C, followed by biotin-conjugated secondary antibody for 1 h at room temperature, streptavidin-peroxidase for 30 min at room temperature, and enzyme substrate (3,3′-Diaminobenzidine, Dako, Denmark). As an additional control, sections were also incubated with phosphate buffered saline (PBS) only, followed by incubation with biotin-conjugated secondary antibody, streptavidin-peroxidase, and enzyme substrate. PBS washes (3 times, 5 min each) were performed during each incubation step. Sections were counterstained with methyl green and visualized by light microscopy (Eclipse 80i, Nikon, Japan). Every tissue section was evaluated by two senior pathologists (Dr. Mi and Dr. Zhang). The kappa statistic was used to assess inter-observer variability with a score of >0.75 indicating excellent agreement. Staining intensity was classified using a numerical scale; grade 0 (none, 0–10 % staining); grade 1 (weak, 10–30 %); grade 2 (moderate, 30–60 %), and grade 3 (strong, over 60 %), with a score of > =2 considered positive of staining with *P. gingivalis* or Lys-gingipain.

### Determination of16S rDNA in fresh ESSC tissue

For each patient, tissues from cancer and adjacent to cancer sites (minimum 3 cm distant) were harvested and used as experimental and internal controls, respectively. Endoscopy biopsy specimens from healthy age- and gender-matched individuals were obtained from normal controls (*n* = 30). Tissues were suspended in 500 μl of sterile phosphate-buffered saline, vortexed for 30 s and sonicated for 10 s. Proteinase K (2.5 mg/ml final concentration) was added and the samples were incubated overnight at 55 °C, homogenized with sterile disposable pestle and vortexed. DNA was extracted as described previously [47] and purified by phenol-chloroform extraction. All samples were stored at −20 °C until further analysis. For amplification, DNA concentrations were adjusted to 20 ng/ml. 16S rDNA samples were amplified as described previously [47] using *P. gingivalis* specific and universal 16S rDNA primers (*P. gingivalis* 16S rDNA primer sequences were: 5′ AGGCAGCTTGCCATACTGCG 3′ (forward) and 5′ ACTGTTAGCAACTACCGATGT 3′ (reverse), and the PCR product size was 404 bp; The universal 16S rDNA primer sequences were 5′ GATTAGATACCCTGGTAGTCCAC 3′ (forward) and 5′ CCCGGGAACGTATTCACCG 3′ (reverse), and the PCR product size was 688 bp). PCR reactions were performed at 95 °C for 5 min, followed by 30 cycles of denaturation at 95 °C for 1 min, annealing at 52 °C for 1 min, and elongation at 72 °C for 1 min with final elongation at 72 °C for 5 min.

### Statistical analysis

All statistical analyses were performed by SPSS statistical package, version 17.0 (SPSS Inc., Chicago, IL, USA). Pearson’s contingency coefficient was used to test for the association between the immunohistochemical staining levels of *P. gingivalis* and Kgp. Correlations between the presence of *P. gingivalis* and clinicopathologic factors were analyzed by ANOVA or Chi-square test, as appropriate. Overall survival was estimated using the Kaplan-Meier method and the log-rank test for comparison. Multivariate analysis was performed to examine if *P. gingivalis* presence was an independent prognostic factor using the Cox proportional-hazards regression model. *P* values of ≤ 0.05 were considered to be statistically significant.

## Additional files

Additional file 1: Figure S1.
*P. gingivalis* 16S DNA in esophageal epithelium. PCR with specific primers for *P. gingivalis* (upper), and a universal primer (lower). Representative images of *P. gingivalis* PCR products from several pairs of cancerous (lanes 2, 4, 6, 8) and adjacent fresh biopsy tissues from ESCC patients (lanes 3, 5, 7, 9), and normal biopsy tissues as a control (Lane 10). Lanes1 and 11 are molecular size markers. (PDF 215 kb) Additional file 2: Table S1. Concordance between the immunohistochemistry of *P. gingivalis* whole antigens and RT-PCR of *P. gingivalis* 16S rRNA in the cancerous tissue from patients with ESCC. (PDF 43 kb)

## Abbreviations

ESCC: Esophageal squamous cell carcinoma
BE: Barrett’s esophagus
Kgp: Lysine-gingipain
TNM: Tumor-node-metastasis
STAT3: Signal transducer and activator of transcription
JAK2: Janus kinase 2
GSK3β: Glycogen synthesis kinase 3 beta
NDK: Nucleoside diphosphate kinase
PAR: Proteinase-activated receptor
CDK: Cyclin-dependent kinase
MMP: Metalloproteinase
OSCC: Oral squamous cell carcinoma
