# Supplementary material for: Presence of Porphyromonas gingivalis in esophagus and its association with the clinicopathological characteristics and survival in patients with esophageal cancer
Source: Infect Agent Cancer. 2016 Jan 19;11:3. doi: 10.1186/s13027-016-0049-x (PMC4717526; doi:10.1186/s13027-016-0049-x)
Supplement: Additional file 1: Figure S1. — P. gingivalis 16S DNA in esophageal epithelium. PCR with specific primers for P. gingivalis (upper), and a universal primer (lower). Representative images of P. gingivalis PCR products from several pairs of cancerous (lanes 2, 4, 6, 8) and adjacent fresh biopsy tissues from ESCC patients (lanes 3, 5, 7, 9), and normal biopsy tissues as a control (Lane 10). Lanes1 and 11 are molecular size markers. (PDF 215 kb) [file 13027_2016_49_MOESM1_ESM.pdf]

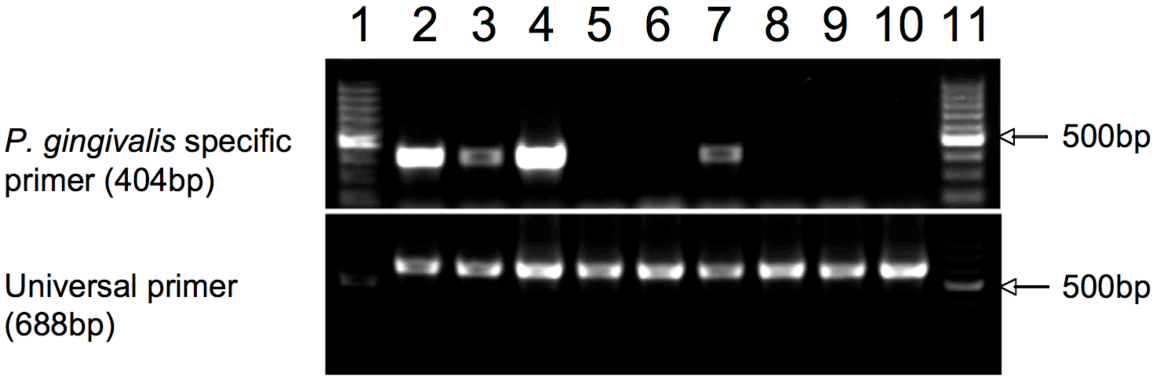

Figure S1

P. gingivalis 16S DNA in esophageal epithelium. PCR with specific primers for *P. gingivalis* (Upper), and a universal primer (Lower). Representative images of *P. gingivalis* PCR products from several pairs of cancerous (lanes 2, 4, 6, 8) and adjacent fresh biopsy tissues from ESCC patients (lanes 3, 5, 7, 9), and normal biopsy tissues as a control (Lane 10). Lanes1 and 11 are molecular size markers.
