# Supplementary material for: Presence of Porphyromonas gingivalis in esophagus and its association with the clinicopathological characteristics and survival in patients with esophageal cancer
Source: Infect Agent Cancer. 2016 Jan 19;11:3. doi: 10.1186/s13027-016-0049-x (PMC4717526; doi:10.1186/s13027-016-0049-x)
Supplement: Additional file 2: Table S1. — Concordance between the immunohistochemistry of P. gingivalis whole antigens and RT-PCR of P. gingivalis 16S rRNA in the cancerous tissue from patients with ESCC. (PDF 43 kb) [file 13027_2016_49_MOESM2_ESM.pdf]

Supplemental table 1 Concordance between the immunohistochemistry of *P. gingivalis* whole antigens and RT-PCR of *P. gingivalis* 16S rRNA in the cancerous tissue from patients with ESCC

Approaches	**PCR** expression		Kappa value	*p* value
	+	-		
**IHC** (+)	67	1	0.882	<0.0001
**IHC** (-)	4	28		
Total	71	29		

* Kappa>0.7 excellent; 0.4-0.7, good; <0.4, poor agreement.
